# Development of modified multi-parametric CT algorithms for diagnosing clear-cell renal cell carcinoma in small solid renal masses

**DOI:** 10.1186/s40644-025-00847-3

**Published:** 2025-02-28

**Authors:** Pengfei Jin, Linghui Zhang, Hong Yang, Tingting Jiang, Chenyang Xu, Jiehui Huang, Zhongyu Zhang, Lei Shi, Xu Wang

**Affiliations:** 1 Department of Radiology, Zhejiang Cancer Hospital, Hangzhou Institute of Medicine (HIM), Chinese Academy of Sciences, Hangzhou, China; 2 Department of Pathology, Zhejiang Cancer Hospital, Hangzhou Institute of Medicine (HIM), Chinese Academy of Sciences, Hangzhou, China

**Keywords:** Clear-cell renal cell carcinoma, Renal mass, Active surveillance, CT, Heterogeneity

## Abstract

**Objective:**

To refine the existing CT algorithm to enhance inter-reader agreement and improve the diagnostic performance for clear-cell renal cell carcinoma (ccRCC) in solid renal masses less than 4 cm.

**Methods:**

A retrospective collection of 331 patients with pathologically confirmed renal masses were enrolled in this study. Two radiologists independently assessed the CT images: in addition to heterogeneity score (HS) and mass-to-cortex corticomedullary attenuation ratio (MCAR), measured parameters included ratio of major diameter to minor diameter at the maximum axial section (Major axis / Minor axis), tumor-renal interface, standardized heterogeneity ratio (SHR), and standardized nephrographic reduction rate (SNRR). Spearman's correlation analysis was performed to evaluate the relationship between SHR and HS. Univariate and multivariate logistic regression analyses were employed to identify independent risk factors and then CT-score was adjusted by those indicators. The diagnostic efficacy of the modified CT-scores was evaluated using ROC curve analysis.

**Results:**

The SHR and heterogeneity grade (HG) of mass were correlated positively with the HS (R = 0.749, 0.730, all *P* < 0.001). Logistic regression analysis determined that the Major axis / Minor axis (> 1.16), the tumor-renal interface (> 22.3 mm), and the SNRR (> 0.16) as additional independent risk factors to combine with HS and MCAR. Compared to the original CT-score, the two CT algorithms combined tumor-renal interface and SNRR showed significantly improved diagnostic efficacy for ccRCC (AUC: 0.770 vs. 0.861 and 0.862, all *P* < 0.001). The inter-observer agreement for HG was higher than that for HS (weighted Kappa coefficient: 0.797 vs. 0.722). The consistency of modified CT-score was also superior to original CT-score (weighted Kappa coefficient: 0.935 vs. 0.878).

**Conclusion:**

The modified CT algorithms not only enhanced inter-reader consistency but also improved the diagnostic capability for ccRCC in small renal masses.

**Supplementary Information:**

The online version contains supplementary material available at 10.1186/s40644-025-00847-3.

## Introduction

Renal masses are commonly detected incidentally on imaging with high frequency, most of which are benign cysts [1]. However, the characterization of solid renal masses (defined as the presence of over 25% enhancing tissue), particularly small solid masses (≤ 4 cm), remains challenging [2, 3]. Benign tumors, mainly Oncocytomas and angiomyolipomas (AML), accounted for up to 20% of small solid masses [4]. Additionally, stage cT1a renal cell carcinoma (RCC) typically exhibits a quiescent clinical course, with infrequent occurrences of local recurrence or metastasis [5]. Consequently, active surveillance (AS) plays an increasingly crucial role in the management of clinically limited renal solid masses [6]. For RCCs, the aggressiveness of the mass (often determined by histologic subtype and pathologic grading) serves as a significant factor driving and influencing the AS program. Clear-cell RCC (ccRCC), being the most prevalent subtype of RCC, also represents the highest risk for metastasis and progression during AS [7, 8].

Currently, pathological analysis following biopsy or surgical resection is the primary modality identifying ccRCC in small solid renal masses [9]. However, biopsy represents an additional invasive diagnostic procedure, with approximately 8% of patients potentially experiencing complications [10]. Moreover, up to 20% of lesions may lack a definitive pathological diagnosis due to inadequate tissue samples, resulting in treatment delays [11]. Non-invasive imaging methods that can predict pathological diagnosis offer reliable information for reducing unnecessary surgeries and AS management decisions [6]. The multi-parameter MRI-based ccRCC likelihood score (MRI-ccLS) incorporates various imaging findings, including the signal characteristics, enhancement patterns, and diffusion-limited conditions, aiming to standardize lesion scoring and predict the probability of a solid mass being ccRCC [12]. The accuracy and inter-observer agreement of MRI-ccLS are moderate but exhibit a high negative predictive value (NPV) [13–15]. The limitation of MRI-ccLS lies in the restricted availability of MRI in addition to the complexity [16] of image postprocessing methods. Under the premise of maintaining the radiation exposure to patients and personnel within reasonable limits, CT scans are less time-consuming and more economical, thereby enhancing patient tolerance in the evaluation of renal masses [17].

Al Nasibi et al. [18] proposed a five-tiered algorithm utilizing multiphasic CT scans to predict the likelihood of ccRCC in small solid renal masses, which revealed significant disparities in mass-to-cortex corticomedullary attenuation ratio (MCAR) and heterogeneity score (HS) between ccRCC and other masses. The Lemieux’s team [19] validated the algorithm externally, demonstrating its NPV for diagnosing ccRCC, as well as its moderate sensitivity and positive predictive value (PPV). However, the HS was subjectively assessed, resulted in the substantial variation in image interpretation among readers (Kappa coefficient was only 0.57), with an agreement coefficient of 0.32 for the overall CT-score in the external validation cohort [19, 20]. Moreover, the aspect ratio, contact range with normal tissue, and washout rate of tumors have been demonstrated to hold promising potential for characterizing malignant lesions and clinical staging [21–23]. Nevertheless, the significance of these morphological and enhancement characteristics in renal masses remains uncertain. Therefore, this study aims to explore an alternative quantitative indicator for the HS, while incorporating more CT features to enhance inter-reader agreement and diagnostic performance within the existing CT algorithm.

## Methods

### Patient cohort

Adult patients (age ≥ 18 years) underwent surgical resection for renal mass were initially searched in the institutional pathology database from January 2019 to December 2023. Patients were excluded for the following reasons: non-renal primary tumors (e.g., metastases, retroperitoneal tumors); confirmed cystic renal masses according to the pathology report; indeterminate pathologic diagnosis. 584 patients were preliminarily identified. The histologic diagnosis of each mass was assigned by a genitourinary pathologist through the pathological evaluation following the WHO renal tumor criteria [24].

**Fig. 1:**
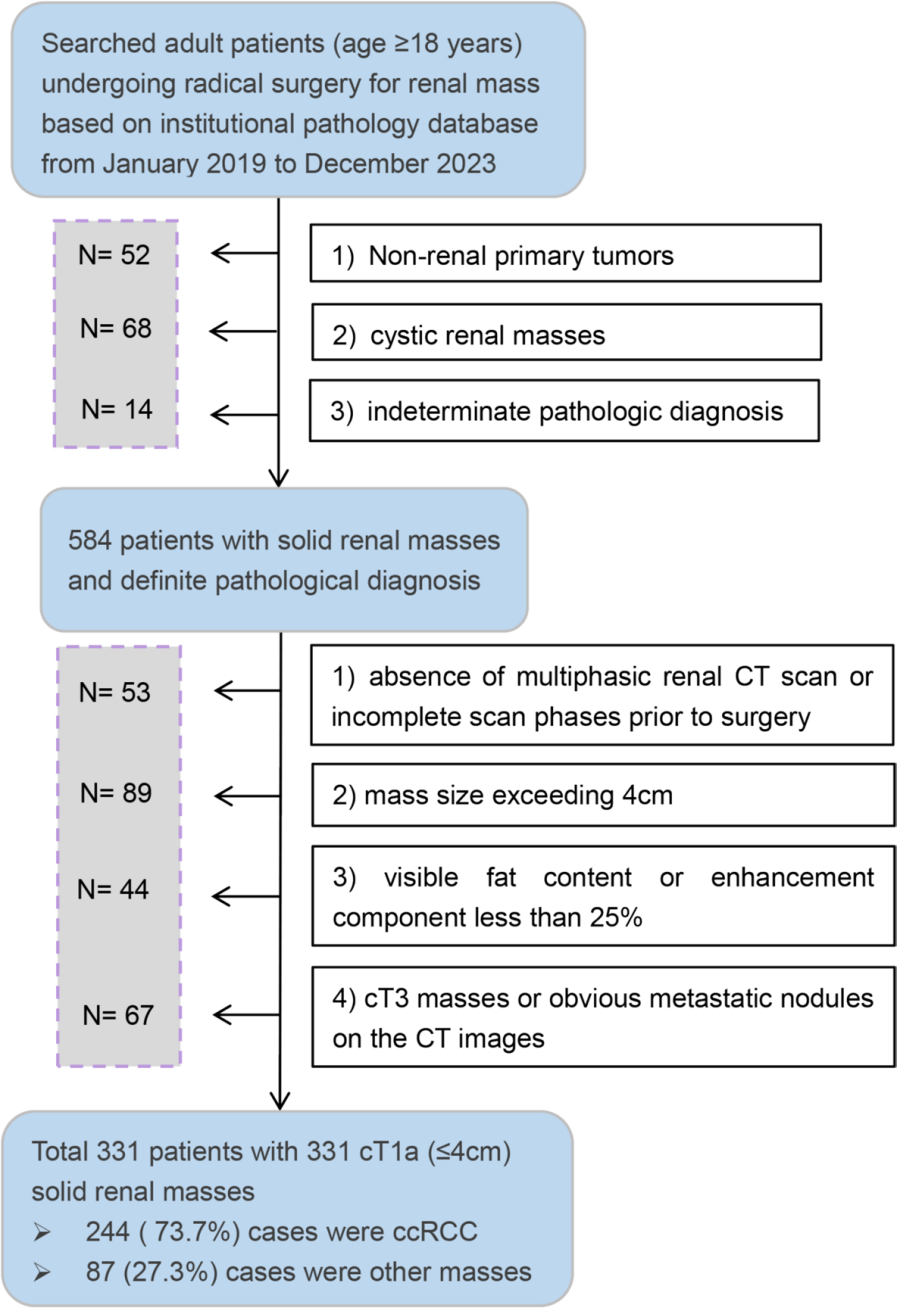
Flow diagram illustrates the criteria for patient inclusion and exclusion in the study

These patients were then reviewed by a radiologist specializing in genitourinary imaging. The investigator perused the CT images, labeled the eligible renal masses, and roughly assessed their basic features. Patients were excluded based on the following criteria: absence of multiphasic renal CT scan or incomplete scan phases prior to surgery; mass size (the mean of axial major axis, axial minor axis, and coronary length) exceeding 4 cm; visible fat content or enhancement component less than 25%; suspicious signs of extra-renal invasion (cT3 masses) or obvious metastatic nodules on the CT images. Ultimately, 331 patients (comprising a total of 331 cT1a renal masses) were retained for further evaluation. Figure 1 illustrates the process of determining the study cohort.

### CT protocol

All patients underwent examination on a 64-slice spiral CT scanner (Siemens Somatom Diagnostics Flash, Siemens AG). The scan included four phases: pre-contrast phase (PCP), corticomedullary phase (CMP), nephrographic phase (NP), and excretory phase (EP). Automatic tube current modulation based on patient weight was utilized, with the following CT scan parameters: tube voltage of 120 V, collimation width of 0.625 mm, scan thickness of 5 mm, and reconstructed thickness of 1 mm. The PCP was collected first, followed by the injection of nonionic contrast agent (Ultravist 370, Bayer Schering Pharma AG) into the vein at a rate of 4.5 ml/s for enhanced scanning. CMP timing was determined using push tracking of a circular region of interest (ROI) placed at the level of the abdominal aortic septum, which was acquired 7 s after the attenuation threshold reached 100 HU (corresponding to 20 s—30 s post-injection), and the NP and EP were obtained at 40 s - 60 s and 90 s - 220 s post-CMP, respectively.

### Image interpretation

Two radiologists (with 3 and 5 years of experience in genitourinary imaging, respectively), who were not involved in the patient screening, performed a comprehensive evaluation of the renal mass based on the annotations provided on the CT images. The pathological results were withheld from them. Prior to reviewing the images, an expert radiologist (with 15 years of experience) organized a training session to ensure that both radiologists followed consistent standards for interpreting CT images using Al Nasibi et al.'s criteria and method [18].

**Fig. 2:**
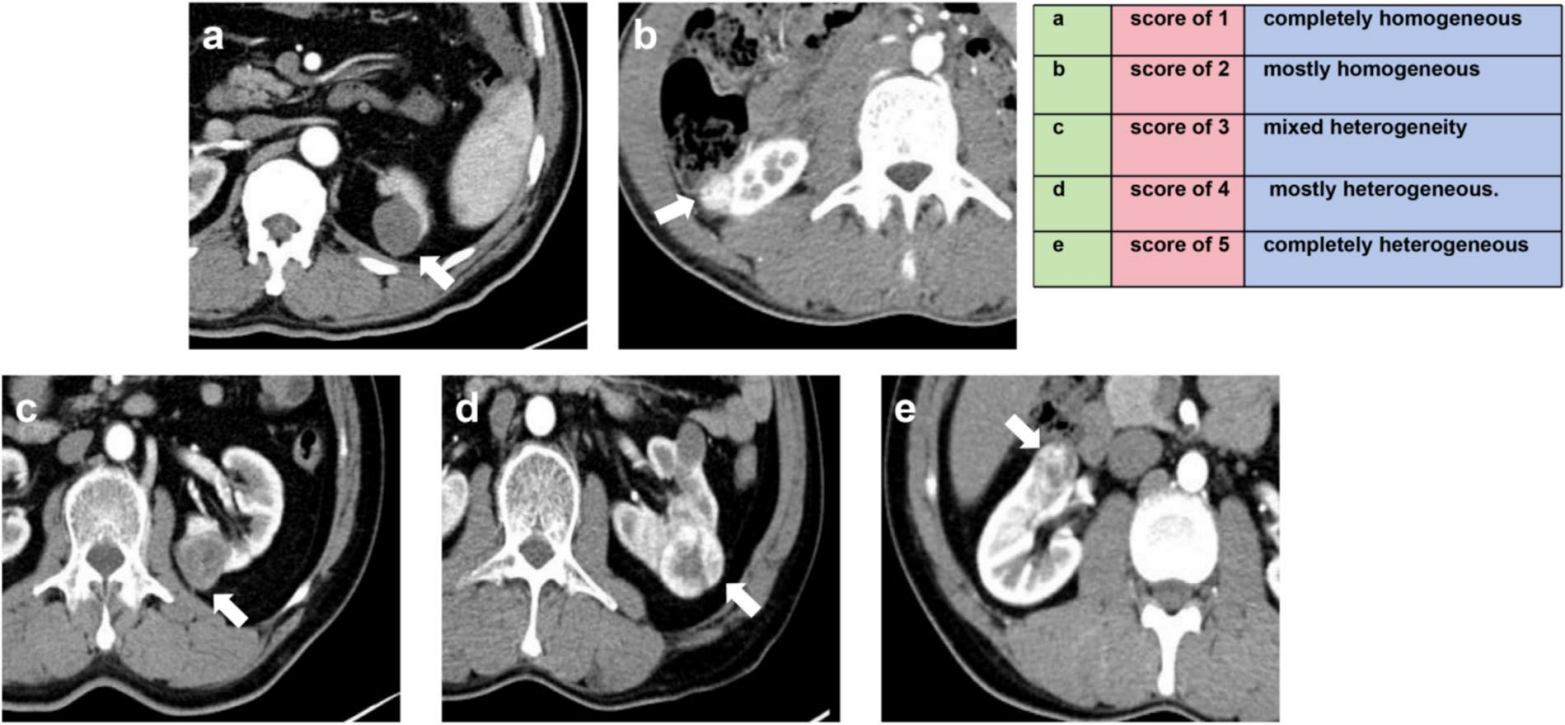
Examples of assessing the 5 grades of homogeneity score based on CT images in axial corticomedullary phase

Subjective assessment: Readers used a 5-point Likert scale to independently assign an HS (based on CMP images) to each mass: 1, completely homogeneous; 2, mostly homogeneous; 3, mixed heterogeneity; 4, mostly heterogeneous; 5, completely heterogeneous (Fig. 2).

Quantitative measurement: To eliminate memory bias, the subjective assessment was performed four weeks later. First, the MCAR (the ratio of mass attenuation value to renal cortex attenuation value at the same slice in CMP) of the mass was recorded. Subsequently, the MCAR was categorized into three graded intervals: 1 = mild (< 0.40), 2 = moderate (0.40 – 0.75), and 3 = intense (> 0.75). Beyond that, we also documented additional CT characteristics: the ratio of major diameter to minor diameter at the maximum axial section (Major axis / Minor axis), the tumor-renal interface, the standardized heterogeneity ratio (SHR), and the standardized nephrographic reduction rate (SNRR). The specific definitions and measurement methods are as follows:

1. Major axis / Minor axis: the major diameter at the maximum axial section of the mass divided by the minor diameter based on CMP images.
2. Tumor-renal interface: the maximum curved surface length of the mass in contact with the renal parenchyma on CMP axial images.
3. SHR: defined as the standard deviation (SD) of the mass attenuation value on CMP images divided by the SD of the aortic attenuation value at the corresponding slice. Placed the ROIs medially on the largest slice of the mass and its adjacent superior and inferior slices. The area of the ROIs accounted for approximately 2/3 of the mass area (based on subjective visual assessment) to obtain the SD of the mass attenuation value. Similarly, standard ROIs with diameter of approximately 5 mm was medially positioned on the same slices of the aorta to determine aortic SD value. The mean of SD across the three slices was used to calculate the SHR.
4. SNRR: defined as the difference between the CMP attenuation value and the NP attenuation value of the mass divided by the CMP attenuation value of the renal cortex at the same slice. The ROI of the mass on the CMP image was copied to the NP image, with any minor discrepancies in ROI registration manually corrected, then measured for NP attenuation.

**Fig. 3:**
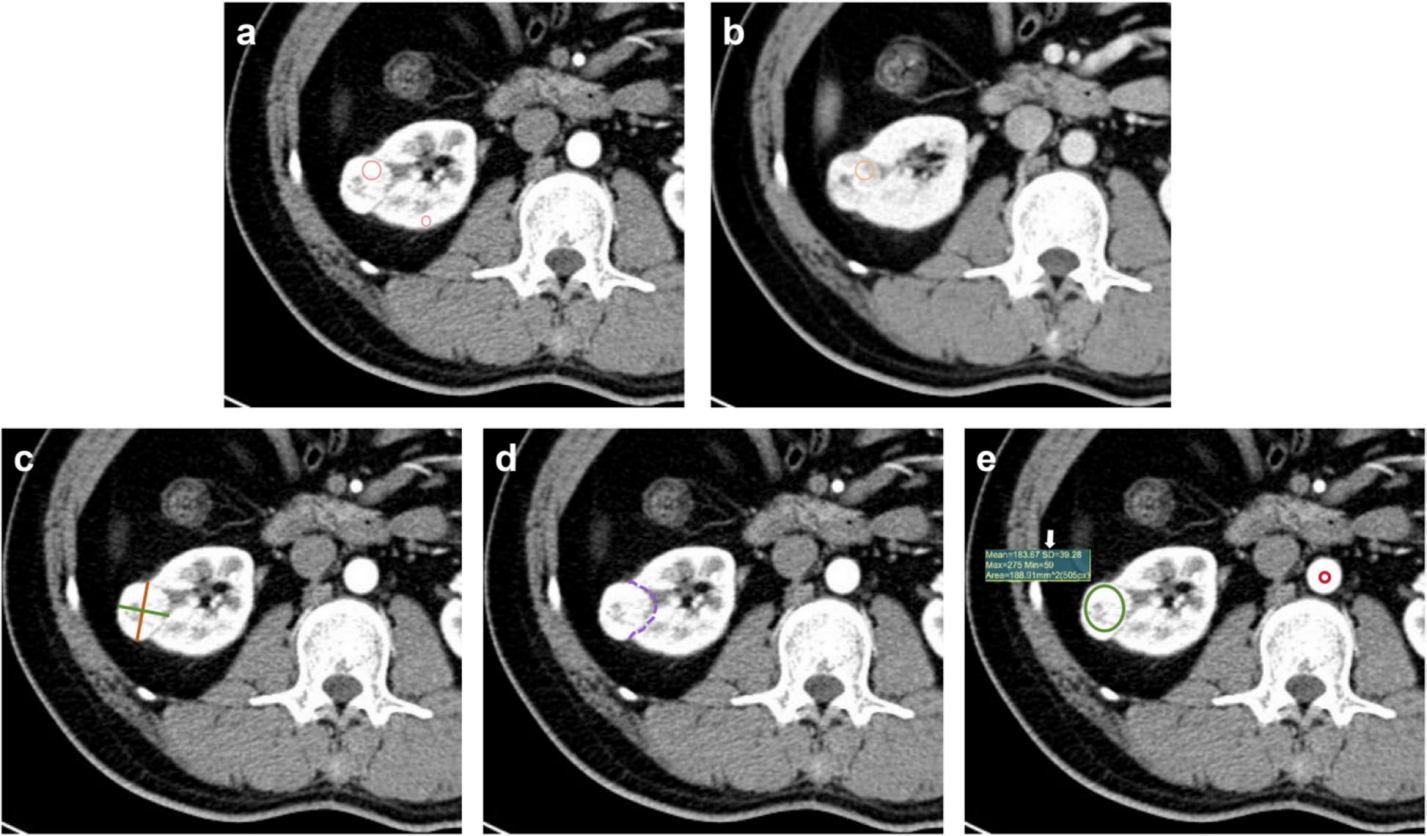
**a** Axial CT images of corticomedullary phase (CMP), placed a region of interest (ROI) in the area where the mass showed the most enhancement (large circle), and another standard ROI in the cortex of the ipsilateral renal (small circle), then recorded the mass-to-cortex corticomedullary attenuation ratio; **b** Axial CT images of nephrographic phase (NP), copied the ROI of the mass on the CMP image to the NP image at the same slice, to attenuation value in NP and determine standardized nephrographic reduction rate; **c** Axial CT images of CMP, measured the maximum major axis and minor axis (mutually perpendicular) of the mass to determine the Major axis / Minor axis; **d** Axial CT images of CMP, recorded tumor-renal interface that the maximum curved surface length of the mass in contact with the renal parenchyma (dotted line); **e** Axial CT images of CMP, place a ROI (green circle) on largest slice of the mass and its adjacent superior and inferior slices, and placed corresponding ROIs (red circle) on the aorta determine to the standard deviation of attenuation value (arrow) and the standardized heterogeneity ratio

Each measurement indicator being measured three times and the average value recorded. A random sample of 50 patients' measurements was examined for consistency. Figure 3 shows the methodology employed for quantifying indicators.

### Statistical analysis

Continuous variables were reported as mean ± SD or median (IQR) and compared using the independent T-test or Mann–Whitney U test assuming normal distribution (Shapiro–Wilk test). Categorical variables were presented as counts (percentages) and compared using a chi-square test. First, alternative indicators for HS and additional indicators with potential diagnostic value were identified. Spearmen correlation analysis was employed to examine the relationship between the HS and the SHR of the mass, and the SHR was categorized into five levels named heterogeneity grade (HG) for further correlation analysis. The optimal cut-off of additional quantitative indicators was determined through ROC curve analysis and converted into binary variables. Subsequently, the binary indicator showing statistically significant difference from the univariate analysis was then separately analyzed with the HG and MCAR using multivariate logistic regression analysis. The original CT-score combined the HS and MCAR based on AI Nasibi et al.'s attribution pathway [18]. Two modifications were made to the CT-score: firstly, replacing HS with HG; secondly, considering the directional impact of independent risk factors identified by multivariate analysis in upgrading (positively correlated) or downgrading (negatively correlated) when the score reached category 3.

ROC curve analysis was employed to assess the performance of both the original and modified CT-scores, while the 1000-bootstrap method was adopted for internal validation. The Fleiss-weighted kappa test was conducted to assess inter-reader agreement for HS, HG, original CT-score and modified CT-score. Inter-reader correlation coefficient (ICC) and Bland–Altman analysis were used to determine repeatability of SHR measurements. Statistical analyses were performed using R language (version 4.1.0). Statistical significance was defined as a *P* value < 0.05.

## Results

### Baseline information

Table 1 summarizes patient demographic characteristics and histological diagnosis. The mean age of the patients was 57.1 ± 10.9 years, with 134 female patients (40.5%) and 197 male patients (59.5%). The proportion of ccRCC was 73.7% (244/331) while other histological diagnoses accounted for 27.3% (87/331). Among non-ccRCC masses, 42.5% (37/87) were malignant tumors, with pRCC being the most common (*N* = 20); 57.5% (50/87) were benign tumors, predominantly fat-poor AML (*N* = 37).

**Table 1:** Patient demographic characteristics and histological diagnosis

Characteristics	Overall
Age, years, mean ± SD	57.1 ± 10.9
Sex, N (%)	
Female	134 (40.5%)
Male	197 (59.5%)
Size, mm, median (IQR)	26.9 (19.1, 32.5)
Histologic diagnosis, n (%)	
Clear-cell RCC	244 (73.7%)
Chromophobe RCC	17 (5.1%)
Papillary RCC	20 (6%)
Fat-poor angiomyolipoma	37 (11.2%)
Oncocytoma	13 (3.9%)

*SD* standard deviation, *IQR* interquartile range, *RCC* renal cell carcinoma

### The HS and SD of attenuation value

**Fig. 4:**
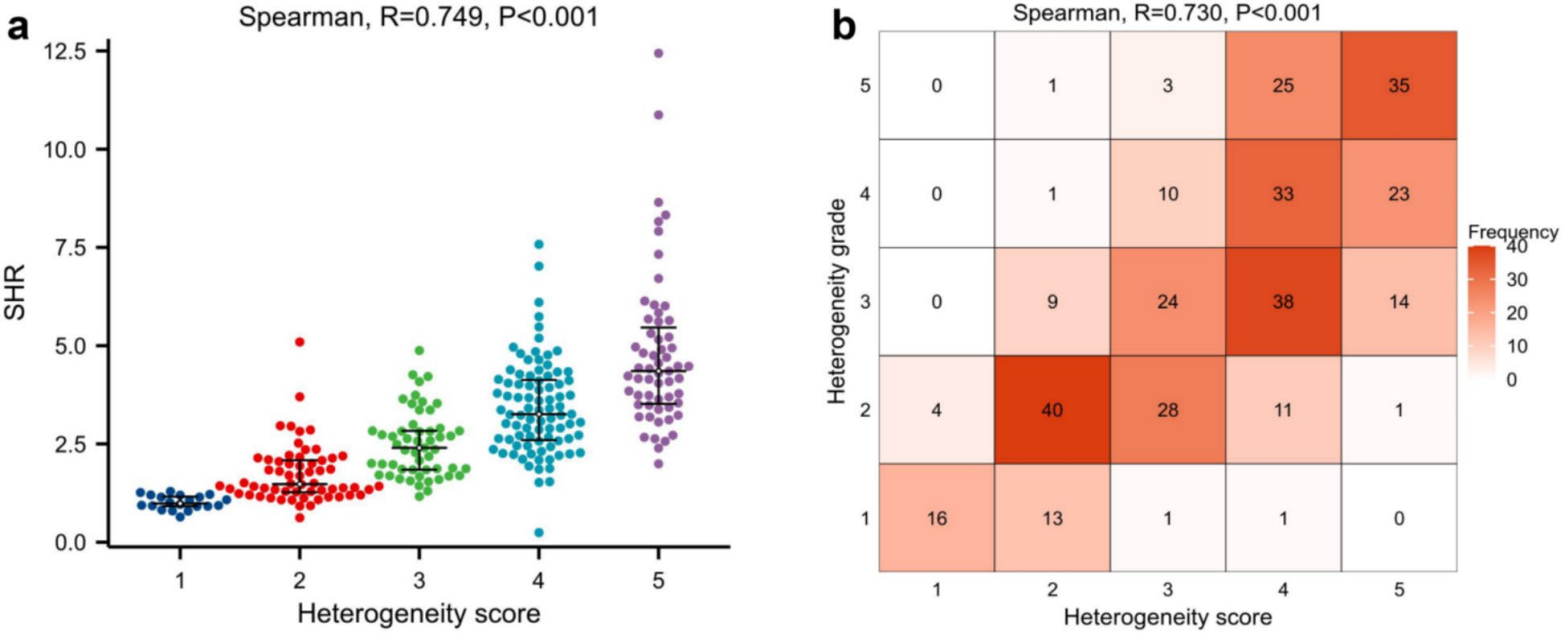
**a** The correlation between SHR and HS (R = 0.749, *P* < 0.001); **b** The correlation between HG and HS (R = 0.730, *P* < 0.001). SHR = standardized heterogeneity ratio, HS = heterogeneity score, HG = heterogeneity grade

The median (IQR) SHR for ccRCC was 3.2 (2.3, 4.2), whereas it was 1.5 (1.2, 2.2) for other renal masses, showing a statistically significant difference (Table 2). SHR was divided into HG with 5 grade intervals: 0 - 1.2 as grade 1; 1.2 - 2.2 as grade 2; 2.2 - —3.2 as grade 3; 3.2 - 4.2 as grade 4; and > 4.2 as grade 5. The Spearmen correlation analysis results (Fig. 4) demonstrated a strong positive correlation between SHR and HS (R = 0.749, *P* < 0.001). Similarly, the HG derived from SHR also showed a strong positive correlation with the HS (R = 0.730, *P* < 0.001). Therefore, SHR (and HG) can be considered a potential alternative indicator for HS.

**Table 2:** Comparison of clinical data and CT variables for ccRCC and other histological diagnoses in small solid renal masses

Characteristics	Clear cell RCC (*N* = 244)	Other renal tumors (*N* = 87)	*P* value
Age,years, median (IQR)	58 (50, 65)	56 (49.5, 62)	0.249^a^
Sex, N (%)			0.026^b^
Female	90 (27.2%)	44 (13.3%)	
Male	154 (46.5%)	43 (13%)	
Major axis / Minor axis, median (IQR)	1.13 (1.07, 1.2245)	1.20 (1.12, 1.38)	< 0.001^a^
Tumor-renal interface,mm, median (IQR)	42.9 (30.7, 52.5)	36.0 (20.9, 49.0)	0.001^a^
Heterogeneity score, n (%)			< 0.001^b^
1	2 (0.6%)	18 (5.4%)	
2	27 (8.2%)	37 (11.2%)	
3	47 (14.2%)	19 (5.7%)	
4	96 (29%)	12 (3.6%)	
5	72 (21.8%)	1 (0.3%)	
Standardized heterogeneity ratio (SHR), median (IQR)	3.2 (2.3, 4.2)	1.5 (1.2, 2.2)	< 0.001^a^
Mass-to-cortex corticomedullary attenuation ratio, median (IQR)	1.10 (0.93, 1.27)	0.63 (0.44, 0.82)	< 0.001^a^
Standardized nephrographic reduction rate, median (IQR)	0.29 (0.13, 0.45)	0.02 (−0.08, 0.12)	< 0.001^a^

*ccRCC* clear cell renal cell carcinoma, *IQR* interquartile range, *Major axis / Minor axis* the ratio of major diameter to minor diameter at the maximum axial section

^a^ Comparisons were performed using the Wilcoxon test

^b^ Comparisons were performed using chi-square test

### Refinement of indicators with supplementary diagnostic value

**Fig. 5:**
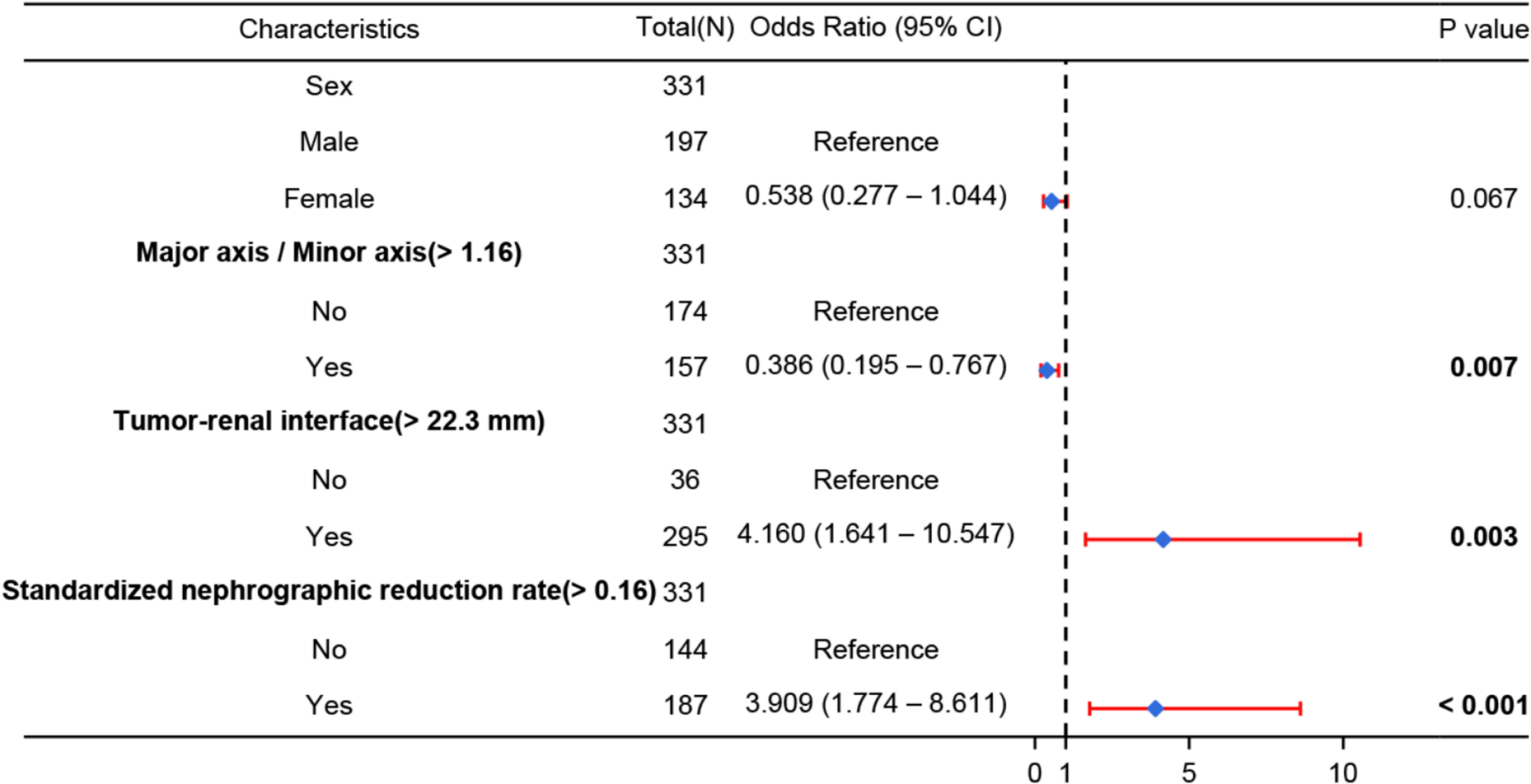
Multivariate logistic regression analysis of forest plot with additional indicators. Major axis / Minor axis (> 1.16), tumor-renal interface (> 22.3 mm), and SNRR (> 0.16) were identified as independent risk factors, which could be utilized to develop separate prediction models for HG and MCAR. SNRR = standardized nephrographic reduction rate, HG = heterogeneity grade, MCAR = mass-to-cortex corticomedullary attenuation ratio

The univariate analysis revealed significant differences between ccRCC and other renal masses in terms of sex, Major axis / Minor axis, tumor-renal interface, and SNRR (Table 2). ROC curve analysis determined the optimal cut-off values for Major axis / Minor axis, tumor-renal interface, and SNRR to be 1.16, 22.3 mm, and 0.16 respectively (Table 3, Figure S1). Multivariate logistic regression analysis showed that Major axis / Minor axis (> 1.16), tumor-renal interface (> 22.3 mm), and SNRR (> 0.16) were independent risk factors associated with the combination of HG and MCAR (Fig. 5). Tumor-renal interface (> 22.3 mm), and SNRR (> 0.16) positively predicted ccRCC (OR = 3.909 and 4.160, all *P* < 0.05). Conversely, Major axis / Minor axis (> 1.16) was negatively correlated with ccRCC (OR = 0.386, *P* = 0.007).

**Table 3:** Determination of the threshold for of additional quantitative indicators included in logistic regression analysis

Variables	Cut-off	Outcome relevance	AUC	95%CI
Major axis / Minor axis	> 1.16	Negative correlation	0.652	0.583 – 0.720
Tumor-renal interface, mm	> 22.3	Positive correlation	0.617	0.544 – 0.691
Standardized nephrographic reduction rate	> 0.16	Positive correlation	0.813	0.763 – 0.863

The optimal threshold was determined by the Youden index of ROC curve analysis

### Modification of CT-score

**Fig. 6:**
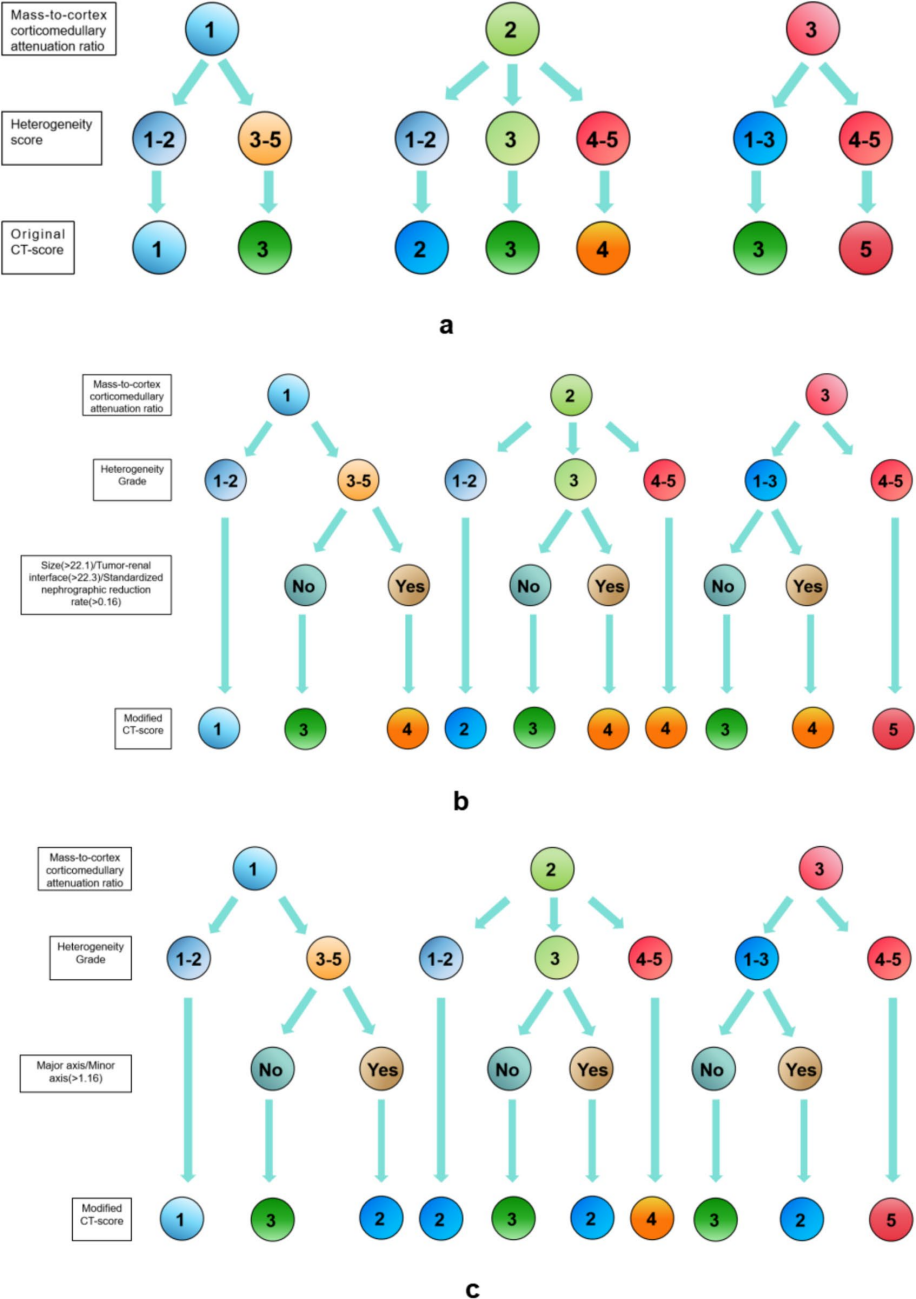
Derivation algorithm for the original CT-score and modified CT-scores. **a** represents original CT-score, mass-to-cortex corticomedullary attenuation ratio (MCAR): 1 = low (< 0.40); 2 = moderate (0.40 - 0.75) greater than 0.75; 3 = intense (> 0.75). Heterogeneity score (HS): 1 = completely homogeneous; 2 = mostly homogeneous; 3 = mixed heterogeneity; 4 = mostly heterogeneous; and 5 = completely heterogeneous. **b** represents modified CT-score 1 and 2 which combined with tumor-renal interface and standardized nephrographic reduction rate (SNRR) respectively; Heterogeneity score (HG): 0 − 1.2 as grade 1; 1.2 - 2.2 as grade 2; 2.2 - —3.2 as grade 3; 3.2 - —4.2 as grade 4; and > 4.2 as grade 5; MCAR: as above mentioned. **c** represents modified CT-score 3: combined with Major axis / Minor axis; HG and MCAR as above mentioned

The original and modified CT-scores are shown in Fig. 6. Initially, HG was substituted for HS, and the combination method remained consistent with the original CT-score. Upon achieving a combined result of 3, the total CT-score was upgraded to 4 after incorporating tumor-renal interface (> 22.3 mm) or SNRR (> 0.16). The total score was downgraded to 2 based on Major axis / Minor axis (> 1.16), and the rest remained unchanged. The schemes respectively combining tumor-renal interface, and SNRR was defined as modified CT-score 1, modified CT-score 2, and the combination of Major axis / Minor axis was defined as modified CT-score 3.

### Diagnostic efficacy of CT-score for ccRCC and pRCC

**Fig. 7:**
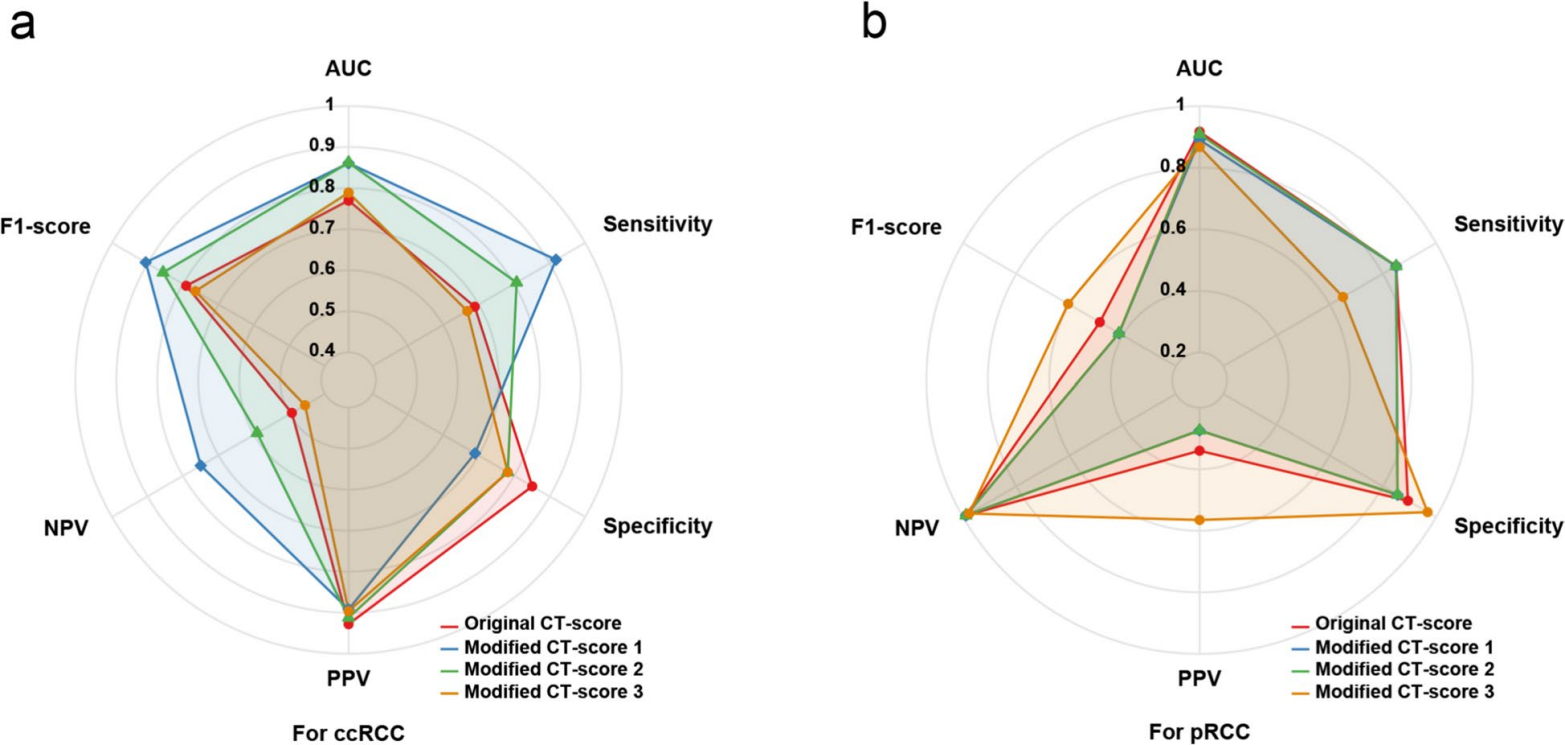
Radar chart illustrating the diagnostic performance of four CT algorithms. **a** specific for ccRCC: Considering the AUC, sensitivity, specificity and F1-score comprehensively, the performance of the modified CT-score 2 demonstrates a relatively well-balanced profile. In contrast, the modified CT-score 3 did not exhibit any significant advantages over the original CT-score. **b** specific for pRCC: The modified CT-score 3 exhibits the highest PPV and F1-score, demonstrating stable overall performance. AUC = area under the curve, PPV = positive predictive value

Table 4 and Fig. 7a summarize the diagnostic performance of each CT-score for ccRCC. The original CT-score had moderate efficacy (AUC = 0.770), while the three modified CT-scores showed moderate to high efficacy (AUCs: 0.861 for modified CT-score 1, 0.862 for modified CT-score 2, 0.789 for modified CT-score 3). After conducting 1,000 resampling and revalidation iterations, the mean AUC values for the original CT-score, modified CT-score 1, modified CT-score 2, and modified CT-score 3 were as follows: 0.790 (95%CI: 0.720 - 0.816), 0.861 (95%CI: 0.811 - 0.905), 0.863 (95%CI: 0.815 - 0.906), 0.789 (95%CI: 0.738 - 0.836). The Delong test showed that the overall diagnostic efficiency was significantly improved after combining tumor-renal interface and SNRR (all *P* < 0.001), while Major axis / Minor axis did not improve diagnostic efficiency significantly (*P* = 0.478). The optimal thresholds for the original CT-score and modified CT-score 1 – 3 were ≥ 4, ≥ 4, ≥ 4, and ≥ 3 respectively. Based on this, the sensitivities of each algorithm were 0.689, 0.918, 0.807 and 0.668 respectively; the PPVs were 0.928, 0.892, 0.912, 0.896; while the F1-scores were 0.791, 0.905, 0.856, and 0.765 respectively. However, the specificity of modified CT-score 1 was only 0.690, compared with a range of 0.782 to 0.851 for the other algorithms.

**Table 4:** Diagnostic performance of original CT-score and modified CT-scores for ccRCC in small solid renal mass

CT algorithms	Cut-off	SEN	SPE	PPV	NPV	F1-score	AUC(95%CI)	*P *value
Original CT-score	≥ 4	0.689	0.851	0.928	0.493	0.791	0.770 (0.722 – 0.817)	-
Modified CT-score 1	≥ 4	0.918	0.690	0.892	0.750	0.905	0.861 (0.815 – 0.906)	< 0.001
Modified CT-score 2	≥ 4	0.807	0.782	0.912	0.591	0.856	0.862 (0.816 – 0.908)	< 0.001
Modified CT-score 3	≥ 3	0.668	0.782	0.896	0.456	0.765	0.789 (0.740 – 0.838)	0.478

*SEN* sensitivity, *SPE* specificity, *PPV* positive predictive value, *NPV* negative predictive value, *AUC* area under the curve, *95%CI* 95% confidence interval

Modified CT-score 1: combined with tumor-renal interface

Modified CT-score 2: combined with standardized nephrographic reduction rate

Modified CT-score 3: combined with Major axis / Minor axis

*P* value: Delong test was used to compare the differences of AUC between modified CT-scores and original CT-score

The diagnostic efficacy of each CT-score for pRCC is summarized in Table 5 and Fig. 7b. Both original CT-score and three modified CT-scores demonstrate excellent performance in diagnosing pRCC. The AUCs were 0.917 (original CT-score), 0.891 (modified CT-score 1) and 0.909 (modified CT-score 2) and 0.868 (modified CT-score 3), respectively, with no statistical significance observed (*P* range from 0.050 - 0.557). The optimal thresholds for original CT-score and modified CT-score 1 – 3 were ≤ 2, ≤ 2, ≤ 2, and ≤ 1 respectively. The sensitivities of each algorithm were 0.850, 0.850, 0.850 and 0.650 respectively, the PPVs were 0.340, 0.274, 0.274 and 0.565 respectively, and the F1-scores were 0.486, 0.414, 0.414 and 0.635. When the scores of the original CT-score, modified CT-score 1, modified CT-score 2 were ≥ 3 (NPV were all 0.989), and the modified CT-score 3 was ≥ 2 (NPV was 0.977), the diagnosis of pRCC could be essentially excluded.

**Table 5:** Diagnostic performance of original CT-score and modified CT-scores for pRCC in small solid renal mass

CT algorithms	Cut-off	SEN	SPE	PPV	NPV	F1-score	AUC (95%CI)	*P* value
Original CT-score	≤ 2	0.850	0.894	0.340	0.989	0.486	0.917 (0.841 – 0.993)	-
Modified CT-score 1	≤ 2	0.850	0.855	0.274	0.989	0.414	0.891 (0.801 – 0.981)	0.216
Modified CT-score 2	≤ 2	0.850	0.855	0.274	0.989	0.414	0.909 (0.828 – 0.989)	0.557
Modified CT-score 3	≤ 1	0.650	0.968	0.565	0.977	0.605	0.868 (0.776 – 0.959)	0.050

*SEN* sensitivity, *SPE* specificity, *PPV* positive predictive value, *NPV* negative predictive value, *AUC* area under the curve, *95%C*I 95% confidence interval

Modified CT-score 1: combined with tumor-renal interface

Modified CT-score 2: combined with standardized nephrographic reduction rate

Modified CT-score 3: combined with Major axis / Minor axis

*P* value: Delong test was used to compare the differences of AUC between modified CT-scores and original CT-score

### Inter-reader agreement

The agreement among readers regarding HS was only moderate, as indicated by a weighted Kappa coefficient of 0.722 (95%CI: 0.595 – 0.850). The ICC for measuring SHR by two radiologists was 0.930 (95%CI: 0.882 – 0.959), and the Bland–Altman plot demonstrated that the measurement error of SHR was −0.04 (95%CI: 0173 – 0.103) mm, with 96% (48/50) of cases falling within the 95% limits of agreement. The consistency of the HG was moderately high, with the weighted Kappa coefficient increased to 0.797 (95%CI: 0.688 – 0.906). Furthermore, the modified CT-score exhibited excellent inter-reader agreement and outperformed the original CT-score (weighted Kappa coefficient 0.935 vs. 0.878). Refer to Figure S2 for details.

## Discussion

Accurately identifying ccRCC in solid renal masses measuring 4 cm or less is a crucial step in ensuring patients receive AS safety [25]. This study not only conducted a retrospective external verification of the original CT-score, but also attempted to adopt quantitative parameters instead of subjective HS and integrated additional imaging features to enhance the diagnostic efficiency of the CT algorithm. We discovered that the calibrated SD of mass attenuation value in CMP could more accurately reflect the degree of nonuniformity and improve consistency in assessing heterogeneity. Two modified CT-scores, which incorporate tumor-renal interface, and SNRR, significantly improve the diagnostic efficiency for ccRCC compared to the original CT-score. When using a threshold of category 4, the detection rate and F1-score for ccRCC was higher. In addition, including an additional indicator did not significantly decrease diagnostic efficacy for pRCC. Therefore, the modified CT-scores show promising potential in guiding biopsy decision-making and management plans for patients with small solid renal masses.

In this study, the optimal threshold for diagnosing ccRCC with original CT-score was ≥ 4, but the corresponding sensitivity was only moderately low, which aligns with the results of the primary study and subsequent validation (overall sensitivity ranged from 0.57 to 0.78) [18–20]. Nevertheless, our assessment of the original CT-score yielded a significantly higher PPV of 0.928 compared to previous studies where PPV ranged from 0.59 to 0.79. This discrepancy may be attributed to readers' thorough understanding of assessment criteria and example images in relevant literature under the expert radiologist guidance prior to utilizing CT-score, leading to a positive learning effect over time. A similar observation has been made regarding MRI-ccLs: Schieda et al.’s study reported a PPV of 76% for diagnosing ccRCC [3], while another study demonstrated an increased PPV of 87%, with comparable sensitivities between both investigations [14].

Among the three modified CT-scores, combining the Major axis / Minor axis did not significantly enhance overall diagnostic efficiency; in fact, the detection rate and prediction precision for ccRCC decreased to some extent, suggesting limited applicability in clinical practice. On the the contrary, the PPV and F1-score of the modified CT-score 3 were superior to those of other CT-scores, suggesting a potentially more reliable performance in predicting pRCC. Although the modified CT-score incorporating tumor-renal interface achieved the highest sensitivity (0.918), it came at the cost of a substantial decrease in specificity (0.690). The high prevalence of ccRCC in this study may falsely inflate the diagnostic efficiency of this protocol, potentially leading to excessive biopsies or treatments if implemented in a real clinical setting. The modification, combined with SNRR, not only improved ccRCC detection recall rate but also maintained high precision compared with original CT-score. The radar chart indicates that the modified CT-score 2 exhibits no significant weaknesses across various evaluation metrics, demonstrating its high comprehensive performance in diagnosing ccRCC. Previous studies have proposed various modifications to CT-score, such as segmental enhancement inversion, high attenuation in PCP, and exclusion of HS while retaining MCAR [19, 20, 26]. However, the final results showed that these modifications did not substantially contribute; therefore, similar adjustments were not made.

In our study, HS and MCAR were significantly different between ccRCC and other histological diagnoses, consistent with the findings of Al Nasibi et al. [18]. Previous reports on mass uniformity mostly relied on subjective judgment from radiologists, leading to inconsistent results [19, 27, 28]. However, our study revealed a strong correlation between the SHR of the tumor in CMP and visually perceived homogeneity. The histological comparative study conducted by Nguyen et al. indicated that renal masses exhibited the most distinct texture features in CMP [29]; therefore, CMP images were chosen for heterogeneity assessment. The SD of the attenuation value is obtained by calculating the variability within the ROI, reflecting the degree of data dispersion in a given area. In this study, the median SHR of ccRCC was significantly higher than that of other tumors (3.2 vs. 1.5, *P* < 0.001). Wang et al. [30] also observed similar results. They found that ccRCC consistently exhibited significantly higher SHR than fat-poor AML during all enhancement periods. Peng et al., on the other hand, found statistically significant differences between RCCs and fat-poor AML regarding their SHR values (2.7 ± 0.10 vs. 1.9 ± 0.12, *P* = 0.002) [31]. Correlation analysis incorporating texture features that characterize heterogeneity, including energy, entropy, and uniformity, may offer valuable background information and biological foundation for understanding attenuation SD or SHR. Moreover, since most diagnostic workstations can directly provide the ROI's SD value, utilizing it as a measure for quantifying tumor heterogeneity becomes a simple and efficient method.

For quantitative parameters, the original CT-score solely incorporated the MCAR to reflect the enhancement characteristics. In the scoring pathways of the original CT-score, 42.9% (3/7) were ultimately classified as the score of 3, potentially leading to an over-representation of this mass category among all masses. Our results showed that tumors with an original CT-score of 3 accounted for 30% (100/331), while in Lemieux et al.'s study [19], this proportion ranged from 4 to 27% across different readers. Furthermore, there was a considerable percentage of ccRCC within masses categorized as a CT-score of 3, ranging from 29 to 53%. Considering both the uneven distribution of categories and the high proportion of ccRCC in masses with a score of 3, the original CT-score may need further improvements by incorporating additional indicators to stratify the specific category.

In this study, the SNRR may be the most robust additional parameter, representing the extent of mass clearance in the NP compared to the CMP, and essentially capturing the dynamic characteristics of the mass enhancement pattern. The previously established clearance rate threshold for ccRCC was higher than that for pRCC, chromophobe RCC and fat-poor AML (0.37 vs. −0.70, 0.28 and 0.13, respectively) [32]. Qu et al. proposed that using a threshold of less than 42.5 HU for absolute de-enhancement, the diagnostic accuracy was 85.19% for oncocytomas [33]. Owing to the absence of a standardized framework, prior studies employed diverse methodologies for measurement and correction, resulting in various threshold values for the enhancement pattern. Nonetheless, all studies consistently indicate that the washout rate of ccRCC is more rapid compared to other subtypes of tumors, which is consistent with the trend observed in SNRR [34, 35]. The modification protocol in Eldehimi et al.’s study incorporated similar parameter (the arterial-to-delayed enhancement ratio) for predicting ccRCC [26]. They discovered that although a threshold of 1.5 suggested a higher likelihood of ccRCC, integrating it into the CT algorithm did not yield a significant diagnostic improvement. Two reasons may due to this discrepancy: firstly, it could be related to variances in contrast agent concentration or inter-individual difference in vascular properties; secondly, they used the ratio of attenuation in CMP and NP whereas we employed the subtraction between these two values normalized against the renal cortex. Additionally, we converted quantitative measures into two categories through thresholds and used them only in specific situations, thereby maintaining the simplicity of the scoring system.

This study has several limitations. First, although our study encompassed 331 patients, a larger sample than previous studies, all data were derived from a single center, making external validation of the modified CT-score unfeasible. To mitigate this issue, we intend to gather at least three external cohorts from multiple centers to comprehensively validate the user-friendliness and generalization ability of the modified CT-scores by assessing the performance of radiologists with diverse fellowship backgrounds. Meanwhile, in accordance with the AS guidelines, we aim to facilitate the clinical application of the modified algorithm for prospective validation. Second, the constitution of masses confirmed by surgical histopathology, excluding some patients who only underwent biopsy or AS, potentially introducing bias in the distribution of mass categories compared to their natural occurrence. Considering the efficacy of close follow-up in detecting invasive tumors and the high diagnostic accuracy of biopsy in identifying subtypes of renal masses [36, 37], it is reasonable to include small renal masses that are not immediately subjected to surgical intervention in the analysis aimed at differentiating high-risk lesions from mild tumors. Finally, all subjective assessments and quantitative measurements were meticulously trained; however, it remains uncertain how readers without specialized experience would perform.

## Conclusion

In this study, we enhanced the predictive efficiency of ccRCC in small solid renal masses by modifying the original CT-score. The substitution of SHR for subjective HS contributes to improving inter-reader consistency. Furthermore, incorporating SNRR may facilitate further stratification of patients.

## Supplementary Information

Supplementary Material 1

## Data Availability

The imaging studies and clinical data used for model development are not publicly available, because they contain private patient health information. Interested users may request access to these data, where institutional approvals along with signed data use agreements and/or material transfer agreements may be needed/negotiated. Derived result data supporting the findings of this study are available upon reasonable requests.

## Abbreviations

RCC: Renal cell carcinoma
ccRCC: Clear-cell renal cell carcinoma
MRI-ccLS: MRI-based ccRCC likelihood score
PCP: Pre-contrast phase
CMP: Corticomedullary phase
NP: Nephrographic phase
EP: Excretory phase
MCAR: Mass-to-cortex corticomedullary attenuation ratio
HS: Heterogeneity score
pRCC: Papillary RCC
SHR: Standardized heterogeneity ratio
SNRR: Standardized nephrographic reduction rate
HG: Heterogeneity grade
