# Supplementary material for: Development of modified multi-parametric CT algorithms for diagnosing clear-cell renal cell carcinoma in small solid renal masses
Source: Cancer Imaging. 2025 Feb 28;25:22. doi: 10.1186/s40644-025-00847-3 (PMC11869432; doi:10.1186/s40644-025-00847-3)
Supplement: Supplementary file 1 — Supplementary Material 1 [file 40644_2025_847_MOESM1_ESM.docx]

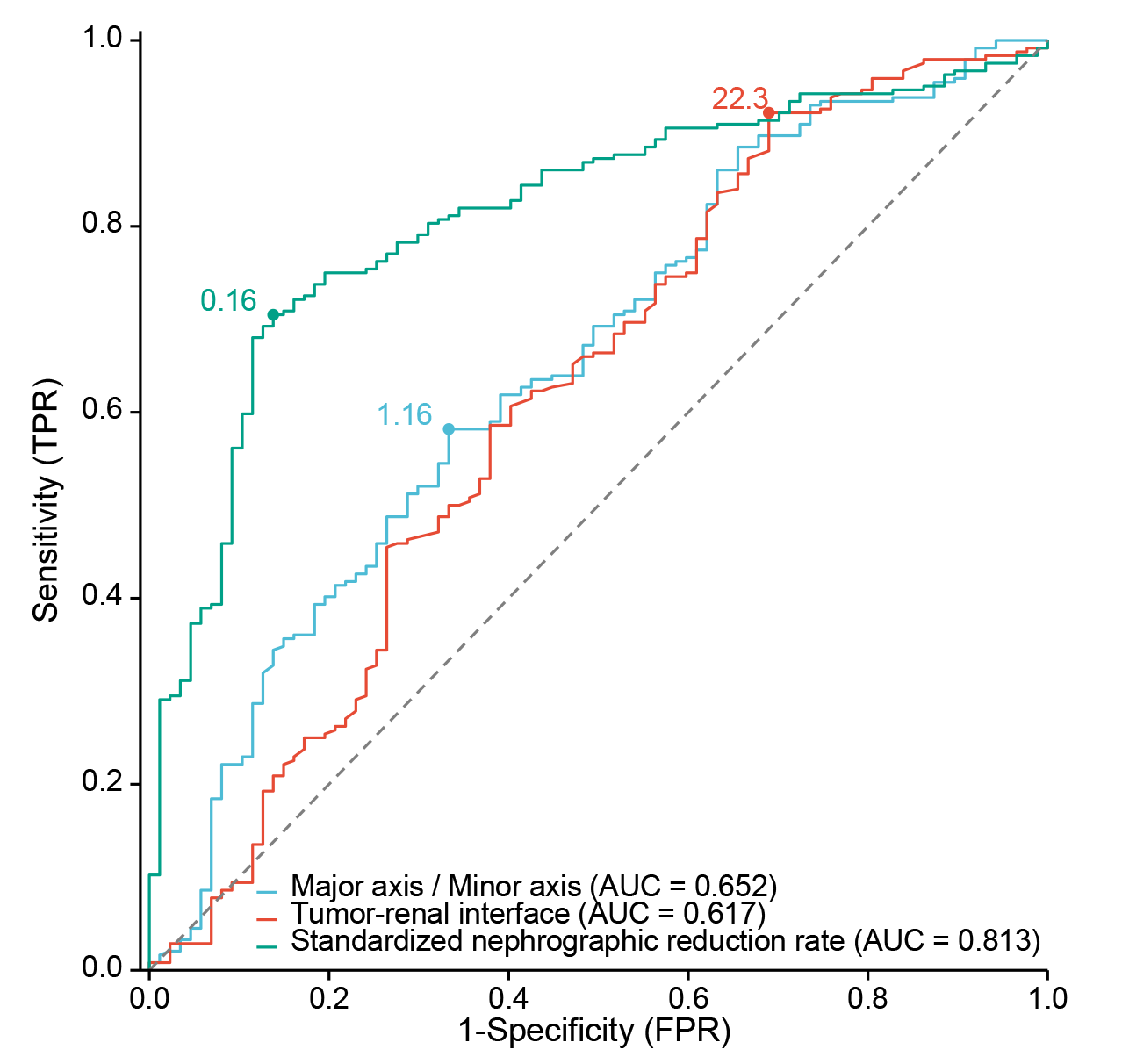


Fig. S1 ROC curve analysis identified the optimal cut-off values for the Major axis / Minor axis ratio, tumor-renal interface, SNRR as 1.16, 22.3 mm, and 0.16, respectively. These cut-off values were subsequently utilized to transform the continuous variables into categorical variables. Major axis / Minor axis ratio = the ratio of major diameter to minor diameter at the maximum axial section; SNRR = standardized nephrographic reduction rate.


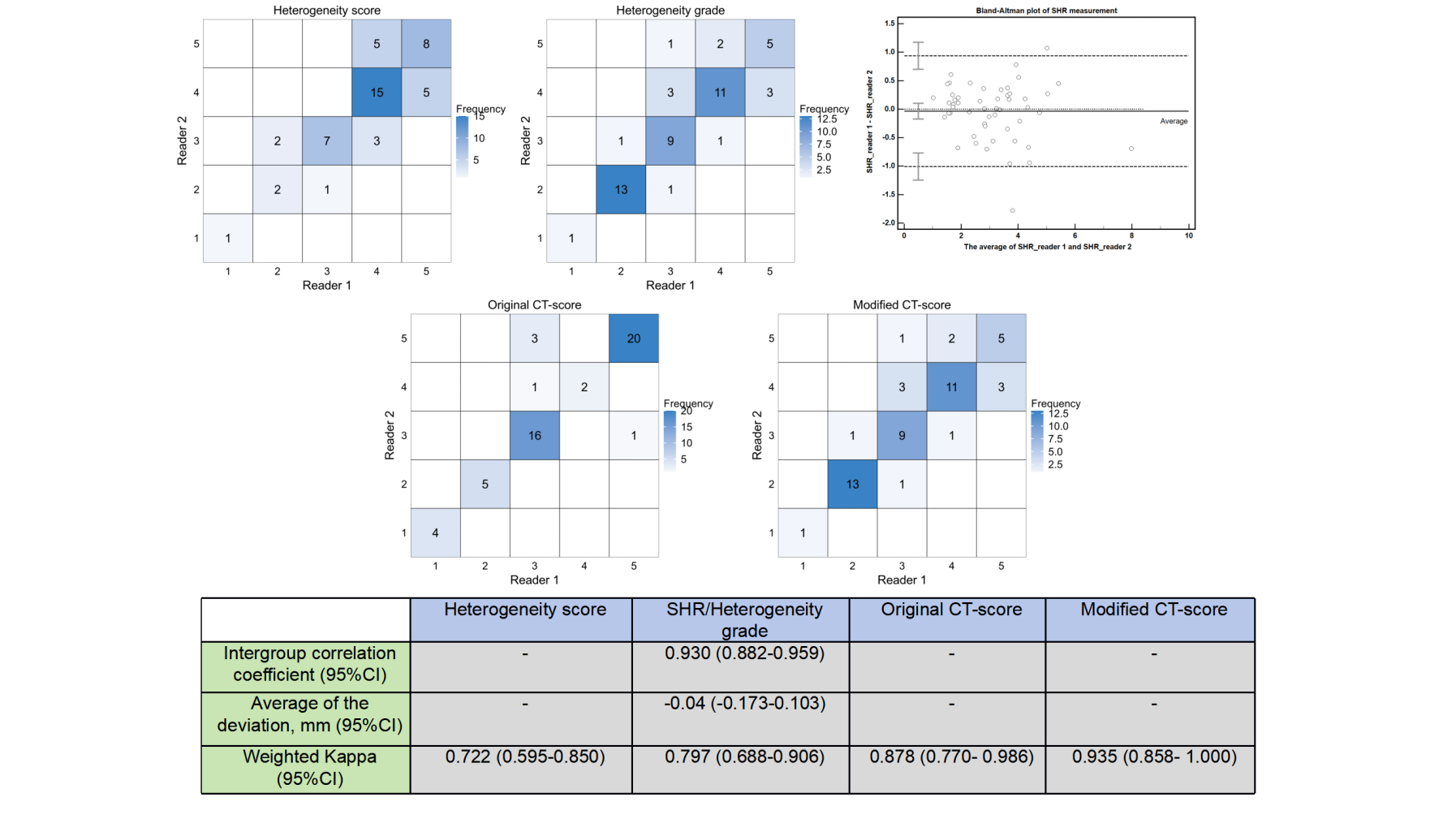


Fig. S2 Inter-observer agreement for SHR measurements, HG and HS assessments, original CT-score, and modified CT-score. The consistency of SHR and HG was found to be higher than that of HS by two readers, thereby enhancing the inter-reader agreement of the modified CT-score.


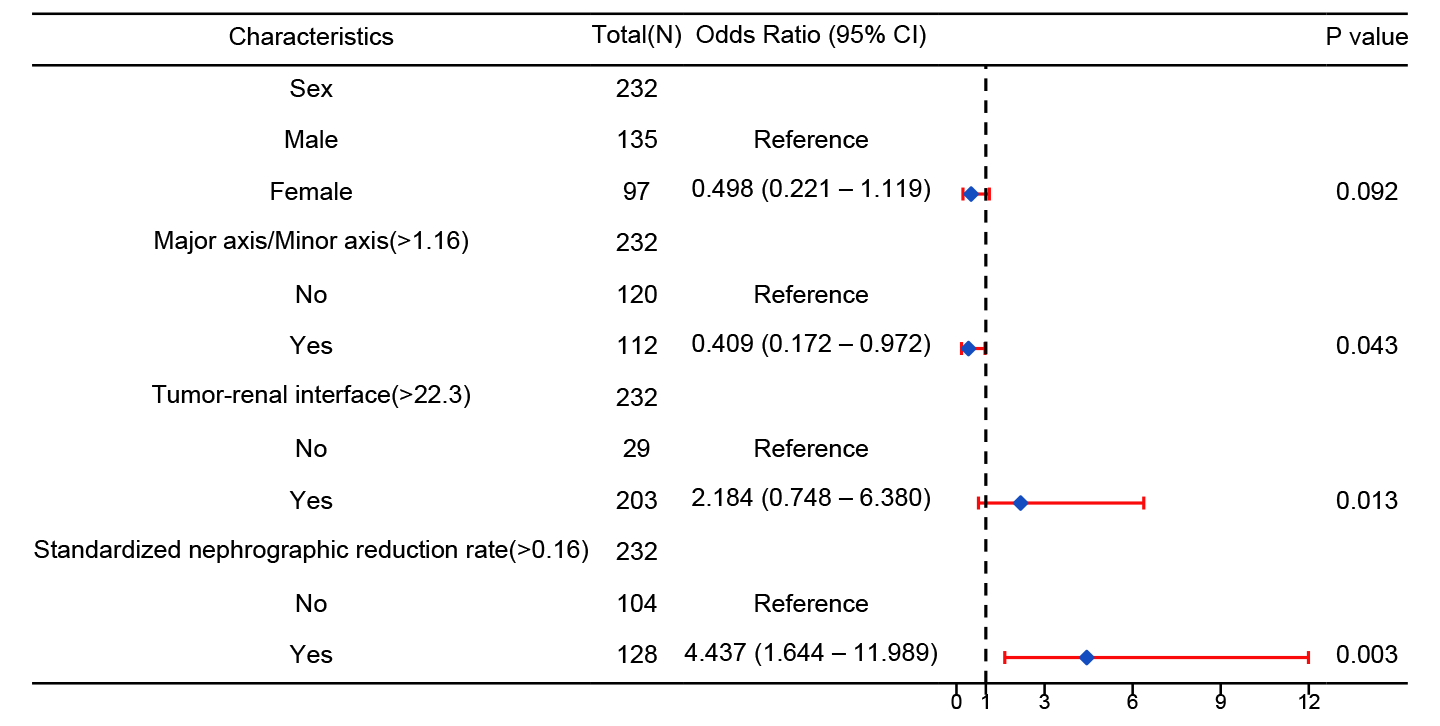


Fig S3 Results of the multivariate logistic regression in the derivation cohort. The original dataset was divided into a derivation cohort (N = 232) and a validation cohort (N = 99) at a 7:3 ratio. In the derivation cohort, ccRCC comprised 73.7% (171/232), while non-ccRCC accounted for 26.3% (61/232). We performed logistic regression analysis on additional quantitative indicators using the method described in the main text. The final independent risk factors identified were Major axis / Minor axis, tumor-renal interface, and standardized nephrographic reduction rate. These resultss were consistent with the original data, with ORs of 0.409 (95% CI: 0.172 - 0.972), 2.184 (95% CI: 0.748 - 6.380), and 4.437 (95% CI: 1.644 - 11.989), respectively. All P-values were less than 0.05.


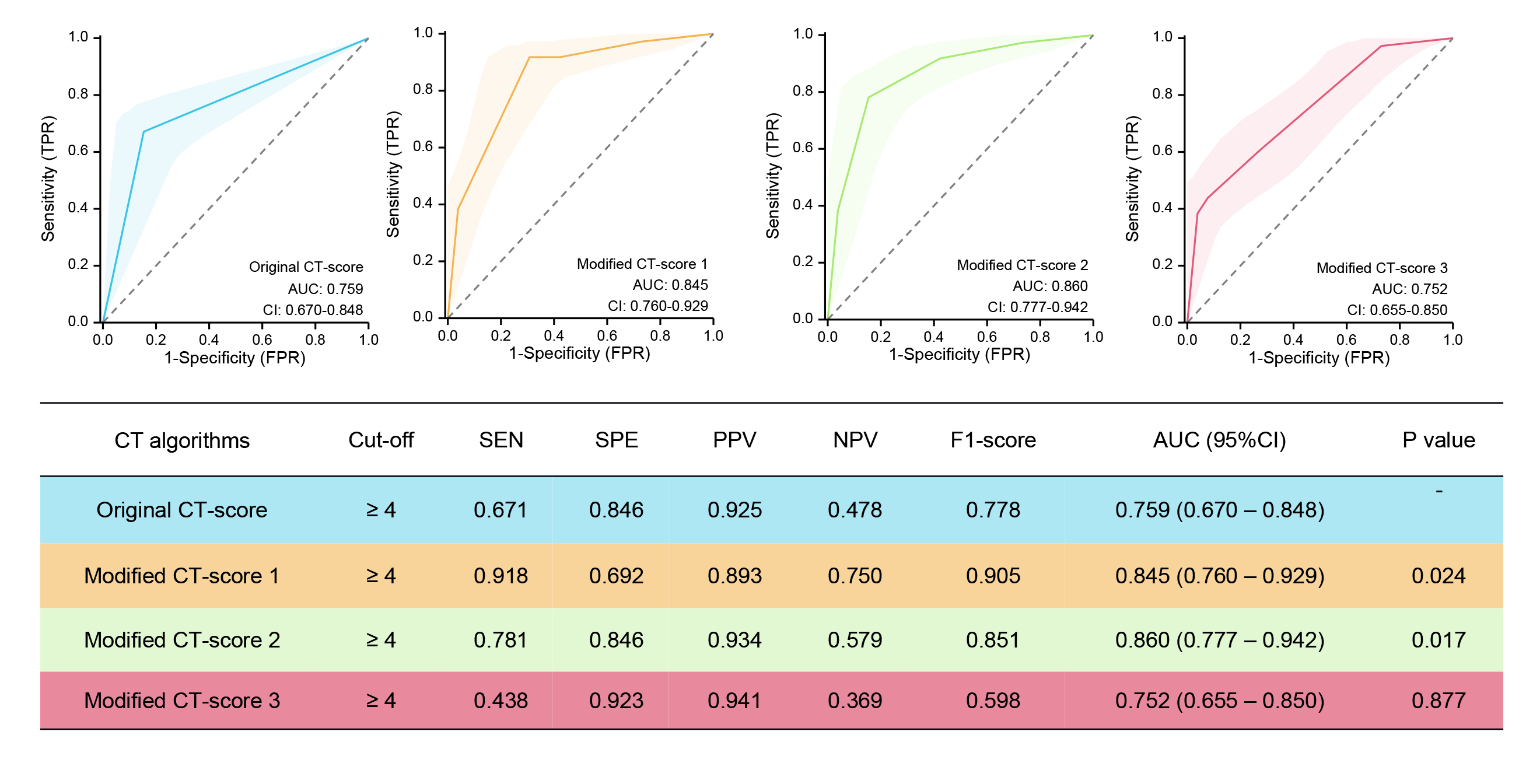


Fig. S4 The performance of the four CT algorithms in diagnosing ccRCC in the validation cohort. In the validation cohort, ccRCC comprised 73.7% (73/99), while non-ccRCC accounted for 26.3% (26/99). Overall, the sensitivity, specificity, PPV, NPV and AUC of the four algorithms in the validation cohort did not exhibit significant differences compared to those in the original cohort. Notably, in the validation cohort, the diagnosticperformance of the modified CT-score 1 and modified CT-score 2 was superior to that of the original CT-score (P < 0.05 for both), whereas the modified CT-score 3 did not demonstrate a significant improvement over the original CT-score (P = 0.877). These findings align with the results observed in the original cohort. ccRCC = clear cell renal cell carcinoma, PPV = positive predictive value, NPV = negative predictive value, AUC = area under the curve. Modified CT-score 1: combined with tumor-renal interface; Modified CT-score 2: combined with standardized nephrographic reduction rate; Modified CT-score 3: combined with Major axis / Minor axis; P value: Delong test was used to compare the differences of AUC between modified CT-scores and original CT-score.
